# Patient and health system delay among TB patients in Ethiopia: Nationwide mixed method cross-sectional study

**DOI:** 10.1186/s12889-020-08967-0

**Published:** 2020-07-17

**Authors:** Daniel G. Datiko, Degu Jerene, Pedro Suarez

**Affiliations:** 1 Challenge TB Ethiopia /Management Sciences for Health Ethiopia, box 1157, code 1250 Addis Ababa, Ethiopia; 2 Management Sciences for Health, Senior Director Infectious Disease Cluster, Arlington, USA

**Keywords:** Tuberculosis, Patient delay, Health system delay, Ethiopia

## Abstract

**Background:**

Effective tuberculosis (TB) control is the end result of improved health seeking by the community and timely provision of quality TB services by the health system. Rapid expansion of health services to the peripheries has improved access to the community. However, high cost of seeking care, stigma related TB, low index of suspicion by health care workers and lack of patient centered care in health facilities contribute to delays in access to timely care that result in delay in seeking care and hence increase TB transmission, morbidity and mortality. We aimed to measure patient and health system delay among TB patients in Ethiopia.

**Methods:**

This is mixed method cross-sectional study conducted in seven regions and two city administrations. We used multistage cluster sampling to randomly select 40 health centers and interviewed 21 TB patients per health center. We also conducted qualitative interviews to understand the reasons for delay.

**Results:**

Of the total 844 TB patients enrolled, 57.8% were men. The mean (SD) age was 34 (SD + 13.8) years. 46.9% of the TB patients were the heads of household, 51.4% were married, 24.1% were farmers and 34.7% were illiterate. The median (IQR) patient, diagnostic and treatment initiation delays were 21 (10–45), 4 (2–10) and 2 (1–3) days respectively. The median (IQR) of total delay was 33 (19–67) days; 72.3% (595) of the patients started treatment after 21 days of the onset of the first symptom. Poverty, cost of seeking care, protracted diagnostic and treatment initiation, inadequate community based TB care and lack of awareness were associated with delay. Community health workers reported that lack of awareness and the expectation that symptoms would resolve by themselves were the main reasons for delay.

**Conclusion:**

TB patients’ delay in seeking care remains a challenge due to limited community interventions, cost of seeking care, prolonged diagnostics and treatment initiation. Therefore, targeted community awareness creation, cost reduction strategies and improving diagnostic capacity are vital to reduce delay in seeking TB care in Ethiopia.

## Background

Tuberculosis (TB) has infected billions and has claimed the lives of millions. Despite the advances in knowledge about the importance of early case finding and treatment, millions of TB cases are being missed by the health system which has led to sustained transmission and increased the pool population at risk of acquiring and developing TB [1, 2]. It is estimated that one active TB case can infect about 10–15 healthy individuals leaving them with 5–10% life time risk of developing active TB [3]. Therefore, programme interventions that ensure early identification of index TB cases and support initiation of prompt treatment with the right dose, quality and duration are required to curb the epidemic.

Effectiveness of the National TB Programme (NTP) depends on its capacity to make early case finding and provision of standard treatment. The diagnostic and treatment services should be decentralized and be accessible to community to seek care. Health seeking behavior is the result of a complex interplay between community awareness, access and availability of the services, cost related to seeking care and sociocultural factors including stigma and beliefs. Factors that affect health seeking behavior lead to delay in seeking care and hence increase TB transmission, mortality and remain a challenge to TB programme performance [4, 5]. Measuring the delay in seeking and getting care is a proxy indicator for good programme performance in reaching and serving the community.

Delay is measured from the time of developing the first symptoms to the time of initiating treatment and is categorized by patient and health facility delay. Patient delay is the time between the onset of the first TB symptom to the time of seeking care while health facility delay is the duration between the first contact with health facility to the time of initiating treatment. Total delay is the sum of TB patient and health facility delays. Treatment delay is measured by the duration from the time of diagnosis to when TB patient started treatment.

Studies reported that delays in seeking care are associated with low awareness about TB, rural residence, high stigma [6], gender [7–10], number and type of facilities visited [11–13], cost of seeking TB care [14], poverty, comorbidity with HIV [15], socioeconomic status [16] and cultural barriers [17]. Total patient delay was more than ten weeks in Sub Saharan Africa [12, 18–20], about seven weeks in Asia - highest in Pakistan and Vietnam nearly doubled [21–24] and about six weeks in Latin America [25]. Though the duration of delay is shorter, it was also reported from low incident states, minority groups and natives due to low index of suspicion about TB [26, 27]. Unfortunately, after patients sought care and arrived at health facilities, there were also further diagnostic and treatment delays [28].

Studies have shown that patient delay contributed more to total delay than did health system delays. However, health system delay has been reported to be longer in smear negative, extra pulmonary TB (EPTB) and among those who visited non TB service providing facilities [29–31]. This could be due to the severity of symptoms TB patients present with that resulted in repeated visits to health facilities, led to protracted diagnostic procedures, and further delayed due to unavailability of diagnostic facilities required to confirm TB diagnosis [32–34].

Ethiopia has been implementing TB prevention and control for about a quarter century. It is successfully treating more than 90 % of enrolled drug sensitive cases [2].

With rapid expansion and decentralization of health services to the community using health extension workers (HEWs), access to TB care has increased and community awareness is expected to also increase.

Studies from Ethiopia reported delays to be associated with educational status, poverty, awareness about TB and accessibility of TB services [35, 36] with higher proportion of health system delay from northern Ethiopia [37]. However, most studies were limited to specific population groups and quantitative designs. In this study, we aimed to measure nationwide delay and the associated factors using a mixed method study using qualitative and quantitative data to understand the context and generate an evidence base to advise policy and decision making at the National TB Programme of Ethiopia.

## Methods

### Study setting and population

Ethiopia is a high TB burden country with a population of more than 100 million. 85% of the population lives in rural areas. Ethiopia is administratively divided into nine regional states and two city administrations. The National TB Programme started DOTS in 1995. Currently 256 hospitals and 3390 health centers provide diagnostic and/or treatment TB services. More than 15,000 health posts provide community based TB services. HEWs are female village residents who completed at least grade ten who received one-year training to provide primary health care to a community of 5000 people. They provide TB services from the health posts under disease prevention and health promotion which includes awareness creation, identifying presumptive TB cases and referral, providing DOT, and retrieving absentees and defaulters [38].

### Study design and sampling

This is a mixed method study conducted from October to November, 2017 in the nine Challenge TB supported regions of Ethiopia that covers 92% of the national population. Challenge TB project is USAID’s global flagship TB control support mechanism designed for 2014 to 2019.

We used a multistage cluster sampling technique to randomly select the zones, districts and kebeles for the study. From six regions and two city administrations, we randomly selected 32 districts and eight sub cities/districts respectively. A health center was randomly selected from each district or sub city with a total of 40 health centers included in the study. From each randomly selected health center, 21 TB patients were consecutively enrolled from each health center. We also conducted qualitative interviews to understand the reasons for delay. The details of the methods are described in a published paper on Knowledge Attitude and Practice about TB in Ethiopia in 2017 [39].

### Operational definition

Patient delay was defined as the time between the first symptoms to date of seeking health care and operationally defined as more than 2 weeks. Diagnostic delay is the interval between first visit to health facilities for TB diagnosis to date of diagnosis for TB. Treatment delay was considered if TB treatment was initiated 1 week or longer after patient was diagnosed with TB. Facility delay is the time between the first visit to treatment initiation. Total delay is the sum of TB patient and facility delay, that is, starting treatment 3 weeks after the onset of symptoms.

#### Quantitative studies

Single population proportion formula was used to estimate the sample size using EDHS 2011 KAP report [7], assuming that 50% of the study participants will have delay of at least 2 weeks to seek TB care. We used design effect of 2 and added 10% to compensate for non-response rate. 844 TB patients were enrolled from 40 health centers.

### Qualitative studies

We conducted 18 Focus Group Discussions (FGD). Two FGDs, one for women and one for men, were conducted in the selected kebeles in the respective regions. The kebele administrators assisted in the identification of participants of the FGDs from the general population.

### Data collection tools and methods

The questionnaires for the study were adopted from the WHO guide for KAP [14]. The questionnaires were initially prepared in English and translated into regional languages and translated back to ensure the quality. FGDs were developed for this study and were conducted in local languages using pretested, semi-structured in-depth interviews (IDI) and open ended topic guides for FGDs. Two data collectors were deployed per site to conduct FGDs and IDIs. 18 FGD sessions and 76 IDIs were conducted and audio recorded. Tablets were used for data collection using a web-based data tool using CSPro software.

### Data management and analysis

#### Quantitative data

Data was extracted from the web-based system and exported to SPSS version 25 for analysis. Mean, median and interquartile range was used to describe the data. Binary logistic and multivariate logistic regression, for independent variables with *p* < 0.25, were used, which includes marital status, wealth index, gender, travel time to health institution, setting and region to construct multivariate model. Addition of other variables like occupation and education compromised model fitness.

#### Qualitative data

Was imported to Open Code software and analyzed using thematic content analysis technique. Direct verbatim and results from the coding and categorization were used to develop the narrative, triangulated and synthesized with the quantitative results [39, 40].

### Data quality assurance

We selected and trained experienced data collectors. Pretested questionnaires were used for data collection. Supervisors were assigned and conducted random data and household checks, and reviewed the questionnaires. The CSPro expert regularly checked for completeness and errors.

### Ethical considerations

Ethical clearance was obtained from the Ministry of Science and Technology. We obtained support letters from the Federal Ministry of Health to the regions and research areas. Ethics review committee approved informed verbal consent to be obtained from the study participants as most of the study participants are from rural and cannot read and write. We obtained informed consent from the study participants.

## Results

### Socio-demographic and economic characteristics of TB patients

A total 844 TB patients were interviewed. The mean (SD) of age was 34 (13.8) years. Of the total study participants, 57.8% were men, 46.9% were the heads of the household, 51.4% were married, 34.7% were illiterate and 24.1% were farmers (Table 1).

**Table 1:** Socio-demographic and economic characteristics of TB patients

Variables (***N*** = 844)	Categories	#	%
Gender	Male	488	57.8
	Female	356	42.2
Age in years^a^	18–24	251	29.7
	25–34	246	29.1
	35–44	164	19.4
	45–54	93	11.0
	55–64	52	6.2
	> 60	38	4.5
Relationship to the head of the HH	Head	396	46.9
	Spouse	189	22.4
	Son/Daughter	222	26.3
	Other relative	33	3.9
	Non-relative	4	0.5
Religion	Orthodox Christian	472	55.9
	Muslim	175	20.7
	Protestant	186	22.0
	Catholic	7	0.8
	Other	4	0.5
Marital Status	Married	434	51.4
	Never married/never lived together	283	33.5
	Divorced/ Separated	66	7.8
	Widowed	57	6.8
	Living together	4	0.5
Educational Status	Not able to read and write	292	34.7
	Read and write only	40	4.7
	Primary	263	31.2
	Secondary	163	19.3
	Above secondary	85	10.1
Occupation	Employed	102	12.1
	House wife	114	13.5
	Farmer	203	24.1
	Daily laborer	103	12.2
	Trader	85	10.1
	Student	109	12.9
	No job/dependent	102	12.1
	House maid	18	2.1
	Other	8	0.9
Wealth Quintile	Lowest	168	19.9
	Second	169	20.0
	Third	175	20.7
	Fourth	163	19.3
	Highest	169	20.0

^a^Range: 18–85 years; Mean (SD): 34 (13.8) years

The mean family size and number of households per sleeping room was 4.5 and 3.6 respectively. Of the study participants, 37.4% had at least one child under 5 years old in their household.

### Patient delay

The median (IQR) patient delay was 21 days (10–45) with signs and symptoms of TB before they first sought health care. 69.9% (575) of the patients sought health care after 2 weeks of onset of symptoms.

### Health system delay: diagnostic and treatment delay

The median (IQR) number of days from the first day of seeking care to diagnosis (diagnostic delay) was 4 (2–10) days. The median (IQR) value of the number of days from diagnosis to treatment initiation was within 2 (1–3) of diagnosis. The median (IQR) for the total days spent between seeking health care and treatment initiation was 6 (3–15) days. However, 44.1% (363) of the patients started treatment after 7 days of first visit. There was significant regional variation of facility delay (*P* < 0.001) as shown in Table 2.

**Table 2:** Factors associated with patient delay among TB patients in Ethiopia

Variables		Delay	No delay	COR (95% CI)	AOR(95%CI)
		# (%)	# (%)		
Gender	Male	340 (70.8)	140 (29.2)	1.12 (0.83–1.51)	1.19 (0.85–1.65)
	Female	235 (68.5)	108 (31.5)	1	1
Marital status	Married	299 (70.0)	128 (30.0)	1	1
	Divorced	44 (68.8)	20 (31.3)	0.94 (0.53–1.66)	1.05 (0.57–1.91)
	Widowed	44 (78.6)	12 (21.4)	1.57 (0.8–3.07)	1.73 (0.85–3.51)
	Not married	188 (68.1)	88 (31.9)	0.92 (0.66–1.27)	0.97 (0.68–1.38)
Wealth	Lowest	119 (75.3)	39 (24.7)	1	1
	Second	109 (66.5)	55 (33.5)	0.65 (0.4–1.06)	0.58 (0.34–0.98)^a^
	Third	129 (74.6)	44 (25.4)	0.96 (0.58–1.58)	0.82 (0.47–1.42)
	Fourth	120 (73.6)	43 (26.4)	0.92 (0.55–1.51)	0.7 (0.39–1.27)
	Highest	98 (59.4)	67 (40.6)	0.48 (0.3–0.77)	0.39 (0.21–0.71)^a^
Setting	Rural	236 (69.4)	104 (30.6)	0.96 (0.71–1.3)	0.6 (0.4–0.89)^a^
	Urban	339 (70.2)	144 (29.8)	1	1
Travel time to health facility	<=20 min	297 (71.1)	121 (28.9)	1	1
	> 20 min	278 (68.6)	127 (31.4)	0.89 (0.66–1.2)	0.84 (0.6–1.17)
Region	Oromia	103 (61.3)	65 (38.7)	1	1
	Amhara	124 (72.9)	46 (27.1)	1.7 (1.08–2.69)	1.62 (1.003–2.63)^a^
	SNNP	139 (85.3)	24 (14.7)	3.66 (2.15–6.23)	3.78 (2.19–6.52)^a^
	Tigray	57 (66.3)	29 (33.7)	1.24 (0.72–2.14)	1.2 (0.67–2.11)
	Benshangul Gumuz	34 (81.0)	8 (19.0)	2.68 (1.2–6.15)	2.4 (1.03–5.6)^a^
	Gambella	13 (46.4)	15 (53.6)	0.55 (0.25–1.22)	0.37 (0.16–0.88)^a^
	Addis Ababa	46 (56.1)	36 (43.9)	0.81 (0.47–1.38)	0.79 (0.43–1.46)
	Dire Dawa	28 (66.7)	14 (33.3)	1.26 (0.62–2.57)	1.17 (0.54–2.53)
	Harari	31 (73.8)	11 (26.2)	1.78 (0.84–3.78)	2.13 (0.95–4.75)

^a^Statistically significant; Delay: seeking care after 2 weeks since the onset of symptoms

### Total delay

The median (IQR) between first onset of symptoms was 33 (range 19–67) days for TB patients and 72.3% (595) of the TB patients initiated TB treatment after 21 days of the onset of the first symptom.

### Factors associated with patient delay

Compared to the poorest, 42% TB patients in the second and 61% TB patients in the fifth wealth quintiles had low odds of being delayed in seeking care after the onset of symptoms. TB patients from rural areas had 40% low odds of being delayed. There was regional variation of delay in seeking care (*p* < 0.05) as shown in Table 2. The median (IQR) travel time from the patient’s home and the health facility was 20 (15–30) minutes (range: 1–120).

The main reasons given for delay in seeking health care were lack of awareness of the severity of the symptoms in 84.4%, seeking care from other providers (drug vendors, traditional or spiritual healers) in 22.8% (40.4% of these visited private drug vendors), inadequate community referral (only 12.5% of the TB patients were initially seen and referred by HEWs) and referral for treatment initiation in 69.7% of the cases. Most family members (61.5%) and patients (59.2%) indicated that they could reduce delay of seeking care. 25.6% indicate that delay could be reduced by the health system (Table 3).

**Table 3:** Reasons why TB patients delaying seeking health care in Ethiopia

Variables		#	%
Reasons for delay in seeking health care	Not aware of the severity of the symptoms	320	84.4
	Fear of rejection/losing job	25	6.6
	Fear that treatment is expensive	32	8.4
	Lack of time	29	7.7
	Difficult access to health center/transportation issue	19	5.5
	Not having a previous satisfactory experience with the health system	20	5.3
	Feeling as if not delayed in seeking treatment for the symptoms	24	6.3
	Others	7	1.8
Seek advice from private facilities and traditional services		188	22.8
	Private drug vendor	76	40.4
	Religious leader	65	34.6
	Traditional healer	39	20.7
	Others	8	4.3
Initially seen by HEW and referred		103	12.5
Health facility of the first diagnosis	In the same health facility contacted first	249	30.3
	Referred from government hospital	422	51.3
	Referred from government health center	50	6.1
	Referred from private health facility	100	12.2
	Referred from NGO health facility	2	0.2
Who do you think can better reduce the delay in the diagnosis and treatment	The patient	487	59.2
	The family	506	61.5
	The health system	211	25.6
	The government	14	1.7
	Others	7	0.9

### Factors associated with delays in seeking care and initiating treatment: qualitative findings

#### Lack of community awareness

Community awareness about TB is increasing within the communities of Ethiopia due to the community-based initiative of using women community HEWs that provide health education in the communities. However, the level of awareness did not reach an adequate level for the community to seek care as early as possible.

“*… The awareness of the community on TB has not reached the expected level” reflecting the inadequacy of their knowledge despite the efforts …*.” *(SNNPR/ woreda TB focal/FGD).*

Most participants reported delayed visit to health institutions after the onset of symptoms. The main reasons were lack of awareness about TB, considering cough to be due to common cold rather than tuberculosis, postponing seeking care until they were seriously ill, fear of screening for HIV, starting self-treatment of cough, and due to household and social responsibilities.

“*… on average, TB patients seek treatment after two to three weeks of coughing …*.” *(Tigray/ HEW).*

*“The problem here is that TB patients delay to go to the health facility simply because they think the cough is due to common cold.” (HEW/BG).*

#### Diagnostic capacity of public health facilities

TB patients usually seek care from public health facilities. However, when they are not diagnosed they tend to visit alternative services. A few participants reported that TB patients visit holy water, traditional healers, religious sites or procure drugs from illegal drug sellers.

*“TB patients first seek care from health centers. If there is no improvement with medical care given at the health center, they seek care from holy water at church or well-known monastery.” (HEW/Amhara).*

“*…*. *There are also some health professionals who sell drugs of the health center to another individual who sell anti TB drugs behind closed doors. For example, there is one individual in.... kebele who sells drugs behind the closed doors. TB patients in this kebele buy anti TB drugs from this illegal person …*” *(Amhara/ FGD).*

#### Cost of seeking care

A few patients reported that shortage of money or cost of seeking care prohibited them from seeking care early from public health facilities.

“*… due to shortage of money some people go to church to treat TB with holy water. They also visit traditional healers.” (FGD/BG).*

#### Seeking care in private facilities

TB services were decentralized to private sectors late, which has now improved. This has led patients to seek care.

*“As it is known previously, TB treatment service was not given by private health institutions. But now, in 2010 there are 10 private health institutions which provide the service after checking them whether they fulfil the criteria to give the service.” (TB-HIV office/Gambella).*

#### Limited access or inadequacy of community TB care

Most participants reported that TB patients have difficulty visiting a health center for daily doses as most health posts do not treat TB. This is worsened by the lack of transportation.

*“...The other point is, all HEWs are not providing DOT service and only 60-70% of the health centers are providing the service which put burden on the local community. Since people are coming from long distances, they default the treatments and that in turn creates a lot of MDR-TB cases in the zone …*.” *(SNNPR/Zone TB focal).*

The number of HEWs in each health post was reported to be inadequate, considering the volume of work they are expected to do.

*“Health extension workers are exhausted with their work because of having multiple workloads in addition to TB package. Due to this problem, the referral linkage from health extension workers to health centers is weak.” (Woreda/TB-HIV officer).*

Inadequate commitment of HEWs to provide TB service to their community and failure to report their performance contributed to delay seeking care and treatment.

*“The most challenging thing is the health extension workers are not working hard in the community. In some kebeles, they often forget to report on the work they have done.” (Woreda TB focal/Gambella).*

#### Poverty

Most participants reported that TB patients are poor and do not benefit from TB services due to the onerous visits and related costs besides the complex socioeconomic problems.

“*… poor people get less nutritional food which exposes them to develop TB; … poor people are discriminated from social life; …*. *poor people have lack of knowledge; …*. *poor people delay visiting health facilities when they have symptoms of TB; …*. *poor people live in crowded houses, etc.” (Amhara region/HC).*

#### Delay in getting diagnosis

Most informants expressed that it takes longer to initiate TB treatment more in cases of EPTB and MDR-TB.

“*… I didn’t receive care early. It was not because of my fault rather it was because the health professionals failed to diagnose the disease. I don’t believe there is good doctor in Ethiopia. I tried my very best by going to private and government health facilities, but it took them very long time to confirm that I have TB …*” *(Dire Dawa/TB patient).*

Shortage of health professionals including laboratory professionals was one of the reasons for delaying diagnosis.

*“Nearly twenty percent of health institutions do not have laboratory technologists.” (TB/HIV officer/RHB).*

*“The problem related to shortage of laboratory professionals is very critical. We don’t know how to solve it.” (Zone/TB focal/SNNPR).*

#### Delay in initiating treatment

Delay in diagnosis contributed to delay in initiating treatment for diagnosed TB cases. This is especially true for EPTB and MDR-TB.

*“I didn’t receive care early. It was not because of my fault rather it was because the health professionals failed to diagnose the disease. I don’t believe there is good doctor in Ethiopia. I tried my very best by going to private and government health facilities but it took them very long time to confirm that I have TB.” (DD/TB patient).*

## Discussion

Early diagnosis and timely initiation of treatment plays a key role in reducing disease transmission, disease severity and risk of death [20, 21]. We report national level delay in seeking diagnosis and initiation of treatment in Ethiopia. Patients delayed seeking care for 21 days and facility delayed diagnosis for 6 days and treatment for 6 days making total delay of 33 days. This is substantiated by qualitative results from patients and health care workers who identified lack of awareness, cost of seeking care and other socioeconomic factors to influence patient delays.

Compared to delay studies conducted in Ethiopia, we report shorter patient delay of 31 days from northern Ethiopia [41], 30 days reported from Hadiya zone in southern Ethiopia [36], 60 days in Addis Ababa [35] and more than 2 months pretreatment duration from Sidama in southern Ethiopia [42], but longer than study from southern Ethiopia that reported 4 days [43]. There is much reduction in the overall delay as a result of increased coverage of health service, better awareness, difference in the study period and changes in socioeconomic conditions. Delays are longer among elderly and patients with EPTB and smear negative TB cases which could be due to the limited diagnostic capacity to detect TB among the elderly, complex diagnostic pathways and delayed health seeking.

Compared to studies from SSA countries, patient delays are shorter in Ethiopia though higher than 2 weeks reported from Cameroon [44] and similar to 3 weeks reported from Lagos, Nigeria [18] and shorter than reports from Uganda, rural Nigeria, and other low incidence states which is in the range of 4–8 weeks [12, 19, 26]. Patients’ residence, age, distance from health facilities, awareness about TB as a curable disease and fear of testing for HIV were the main reasons for delay.

Women had high odds of longer delays compared to men possibly due to higher stigma, lower priority or attention paid to them by the community, socioeconomic problems and domestic responsibilities they shoulder [8, 24]. This could be complex in settings where there is an inadequately decentralized heath system and gender norms favor men for accessing health services. Hence, gender-sensitive TB services should be identified and designed to address such variations in service delivery.

Patients from urban setting had lower odds of shorter delay compared to rural areas [43] mainly due to better awareness about TB, access to services, better education status and socioeconomic condition. However, in urban slums, urban poverty could be worse for TB patients. Contrary to other studies, we report longer delay in urban areas. This could be due to diagnostic pathways related to diagnosis of smear negative and EPTB and HIV [15, 45, 46]. In addition, 85% of the Ethiopian population lives in rural areas and this may have under represented the urban TB patients as we did not estimate the sample size to analyse urban-rural differences.

Due to low awareness about the disease, about the severity and about availability of services, patients ignore TB symptoms and remain at home or visit traditional or private pharmacies. Moreover, visiting health facilities that do not provide diagnostic services, have inadequate technical capacity, and lack diagnostic facilities like chest x-ray, contributes to the delay.

Emerging themes of the qualitative study reported by TB patients, program managers and community showed that lack of awareness, access to informal or illegal drug sellers, inadequate service provision, fear of knowing one is HIV positive, poverty and delay of service provision due to longer waiting time [8, 15, 34] led to delayed TB care.

Patients from high income countries generally have lower delays in seeking care due to the health infrastructure, improved awareness and health seeking. However, a systematic review indicated similarities [47]. This indicates the importance of reviewing case finding strategy and making pro-poor strategies in their respective contexts. Minorities and disadvantaged people in developed countries still have more delays [16].

In resource constrained settings, poverty and inadequate service decentralization complicate patient pathways. Decentralization of services with adequate technical and diagnostic capacity could facilitate early diagnosis. However, stigma, distance and related cost still remain a challenge to the patients to seek diagnosis and initiate treatment [48].

In the era of the End TB Strategy, reducing patient and household costs of seeking care is one of the key components and yet cost of seeking care is high in Ethiopia [43, 49]. Thus, patient insurance mechanisms, waiver services and possibility of reimbursing costs related to seeking TB care could be potential interventions to improve health seeking in resource-constrained settings. Moreover, decentralization of services to the community, early identification of cases and linking to care appear feasible and cost effective options. There is a community-based health insurance system which covers cost of seeking care for poor TB patients. Generally, understanding patient pathway of seeking care is important to program TB services, reduce delay and reduce cost [34, 50].

In the Ethiopian setting, engagement of the HEWs could be a cost effective model (women residing in villages who were trained and employed by the government to provide health care in the community). However, there is limited disclosure to HEWs and their engagement is low compared to the coverage of the HEWs in the country, more than 40,000 HEWs. Generally, mechanisms that reduce the distance between the community, patient and diagnostic units, bringing treatment units in the proximity of the patients at health posts or households and strengthening referral linkages are required to ensure timely diagnosis and prompt initiation of treatment.

The limitation of the study was that it did not consider subnational variations of delay and sample size in the regions was not adequate to measure why delays varied in the regions. Overall delays are likely to be longer since we did analyse the delays by type of tuberculosis. It is likely that smear-negative and extra-pulmonary TB patients would have experienced longer delays due to diagnostic challenges. Patient pathway analysis which could have helped the NTP in designing interventions to improve health seeking is beyond the scope of the study.

## Conclusion

TB patients delay in seeking care remained a challenge, though there is improvement over time. The NTP therefore should increase community engagement to increase awareness about TB, design interventions that reduce cost of seeking TB diagnosis and treatment, and improve intra and interfaculty referral systems to ensure timely diagnosis and treatment initiation. Large scale patient pathway analysis is recommended to understand context-specific factors affecting timely health seeking and design targeted high impact interventions to reduce patient and health system delay in Ethiopia.

## Data Availability

The datasets used and/or analysed during the current study available from the corresponding author on reasonable request.

## Abbreviations

DOTS: Directly Observed Treatment, Short Course
EDHS: Ethiopian Demographic Health Survey
EPTB: Extra Pulmonary Tuberculosis
FGD: Focus Group Discussion
HEWs: Health Extension Workers
HIV: Human Immunodeficiency Virus
IDI: In Depth Interview
NTP: National Tuberculosis Programme
TB: Tuberculosis
WHO: World Health organization
